# The effect of short polyethylene fiber with different weight percentages on diametral tensile strength of conventional and resin modified glass ionomer cements

**DOI:** 10.4317/jced.53550

**Published:** 2017-03-01

**Authors:** Farahnaz Sharafeddin, Seyed-Ali Ghaboos, Zahra Jowkar

**Affiliations:** 1 Professor, Department of Operative Dentistry, Biomaterial Research Center, School of Dentistry, Shiraz University of Medical Sciences, Shiraz, Iran; 2 Dentist, School of Dentistry, Shiraz University of Medical Sciences, Shiraz, Iran; 3 Assistant professor, Department of Operative Dentistry, School of Dentistry, Shiraz University of Medical Sciences, Shiraz, Iran

## Abstract

**Background:**

The aim of this study was to investigate the effect of polyethylene fiber on diametral tensile strength of conventional and resin modified glass ionomer cements.

**Material and Methods:**

60 specimens in 6 groups (n=10) were prepared. In group 1 conventional glass ionomer (Fuji GC) and in group 2 resin modified glass ionomer (Fuji LC) were as control groups. In group 3 and 4 conventional glass ionomers mixed with short polyethylene fibers in proportion of 1 wt% and 3 wt%, respectively. In fifth and sixth groups, resin modified glass ionomer and short polyethylene fibers were mixed in 1 and 3% wt, respectively. Samples were prepared in a round brass mold (6.5×2.5 mm). After thermo-cycling, the diametral tensile strength of the specimens were tested and data were analyzed with ANOVA and post-hoc tests (*p*<0.05).

**Results:**

Diametral tensile strength of both conventional and resin modified glass ionomer cements increased after mixing with polyethylene fiber (*p*<0.001). Also, reinforcement occurred as the mixing percentage increased from 1% wt to 3% wt in either conventional and resin modified glass ionomer (*p*<0.001).

**Conclusions:**

The polyethylene fiber was shown to have a significant positive influence on diametral tensile strength of two types of glass ionomers.

** Key words:**Conventional glass ionomer, diametral tensile strength, polyethylene fiber, resin modified glass ionomer.

## Introduction

Glass ionomers were introduced due to their unique advantages such as micro-chemical adhesion to tooth structures, their mild pulpal irritation and biocompatibility, but due to some weak mechanical properties, they were not used in restorations of stress bearing areas (1). It was shown that low flexural strength and high abrasiveness of glass ionomers have limited their clinical use especially in posterior teeth restorations (2).

In general, glass ionomer is set by formation of a silica-hydrogel based on acid-base reactions between glass ions and a polyacrylic acid. The structural defects of a glass ionomer act as areas to accumulate physical stresses (3,4). Efforts have been made to add an enforcing phase to chemical structure of either glass part or the polyacrylic acid portion (5,6).

Yli-Urpo *et al.* investigated the effect of bioactive glass (BAG) on reinforcement of resin-modified glass ionomers by adding bioactive glass to resin-modified glass ionomer powder in 10 and 30% wt. They showed that by an increase in weight percentage of BAG, there would be a decrease in the compressive strength (7). However, it was demonstrated that incorporation of nanocrys-talline calcium deficient hydroxyapatite to the commercial GIC enhances the compressive strength of the resulting cements (8).

Different types of fibers such as carbon, glass and polyethylene fibers were used to strengthen the dental materials (9). In 2005, it was shown that a combination of short fibers (length: 2-3 mm) would lead to a composite resin with a significant increase in compressive strength, flexural strength and static load-bearing (10).

It has been reported that short glass fibers (3 and 5% wt) played the role of small bridges between cracks and lead to an increase in diametrial tensile, hardness, flexural strength of the conventional glass ionomer (11). Lohbauer *et al.* used 20% vol short glass fiber to reinforce conventional glass ionomer and reported an improvement in flexural strength and the compression strength (12). Kobayashi *et al.* used 60% vol. glass fibers [length: 9.7±2.1 µm] for reinforcement of glass ionomer cements (13). In both recent studies, the glass fibers was made in the same composition as that of the fluoro-alumino-silicate in the conventional glass powder because assumed to be more effective (12,13). In 2003, short glass fibers of 580 µm length and the composition of SiO2-Al2O3-CaF2-Na3AlF5 were used to reinforce glass ionomers and reported an improvement in flexural and compressive strength. However, it was shown that polyethylene fibers have more effect on flexural strength of conventional and resin modified glass ionomers in comparison to glass fibers (14). Therefore, this study was undertaken to investigate the effect of the mixture of polyethylene fiber and glass ionomer cements on diametral tensile strength.

## Material and Methods

**Figure 1:**
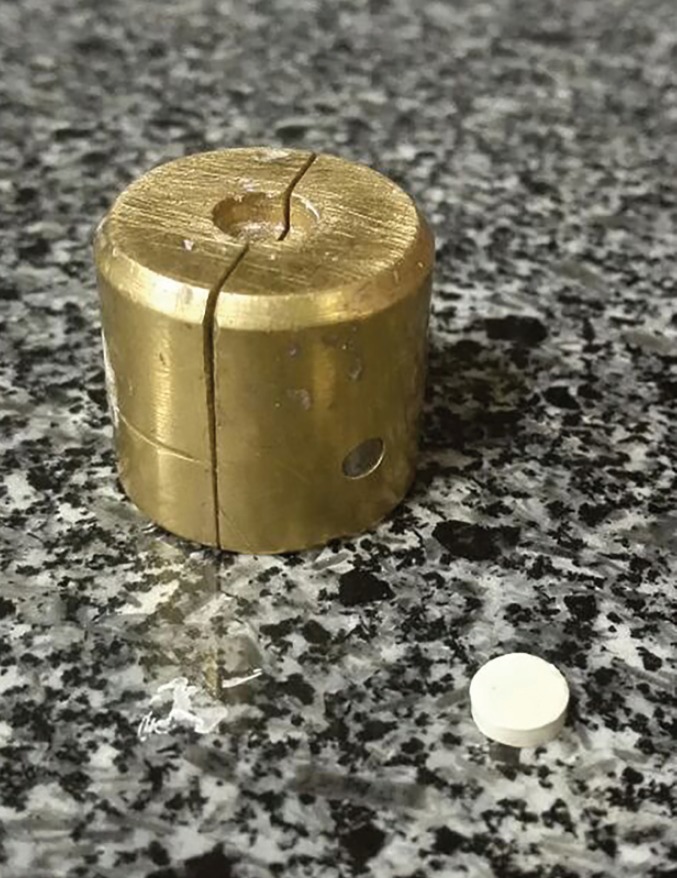
Split mold and specimen.

**Figure 2:**
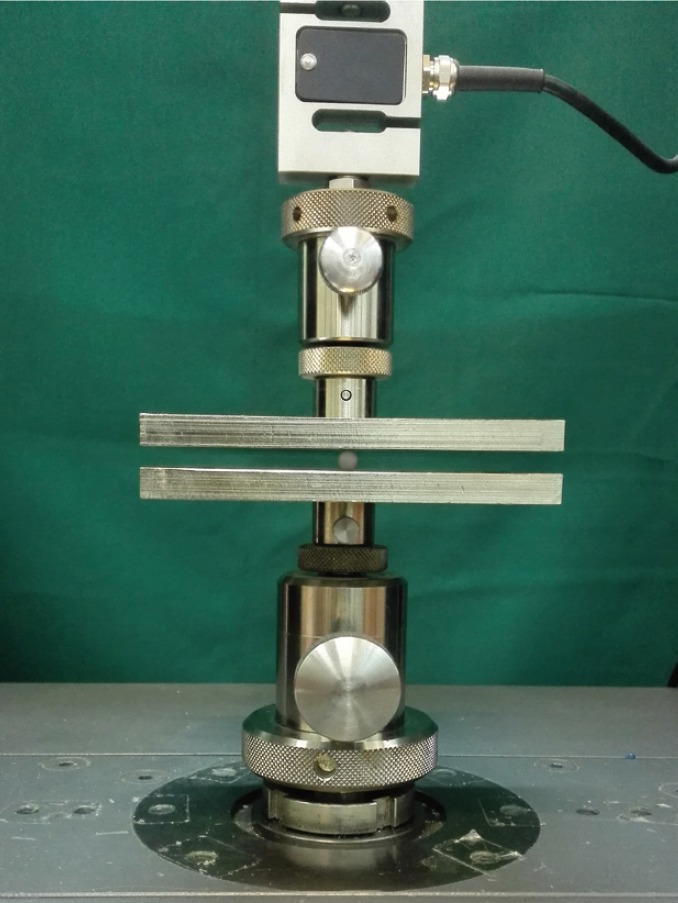
Testing the specimen in universal testing machine.

In this experimental study six groups each containing 10 specimens were enrolled. The first and second control group were considered as conventional glass ionomer group (Fuji GC, Chicago, IL, USA) and resin modified glass ionomer group (Fuji LC, GC, Chicago, IL, USA), respectively. The second control group was cured with an LED unit (Elipar Freelight, 3M ESPE,Germany) with a light intensity of 890 mW/cm² for 20 sec. The third and fourth groups were conventional glass ionomers coupled with short polyethylene fibers in proportions of respectively 1 and 3% wt. In the fifth and sixth groups, resin modified glass ionomer and short polyethylene fibers were mixed in 1 and 3% wt, respectively. The samples were prepared in a round brass mold fig. 1 (diameter: 6.5 mm, height: 2.5 mm) according to the manufacturer’s instruction. Polyethylene fiber was cut to pieces of 1 mm length by a surgical knife of dentistry (blade #15). Glass powder in the needed amount to fill a mold (two scoops for conventional and one for resin modified glass ionomer) was mixed with polyethylene fibers in the desired weight (1 and 3% wt) in empty amalgam capsules (Doumat Amalgamator, Essen, Germany) in amalgamator for 50 seconds. Weight adjustment was carried out using an electronic scale (Precision Health Scale, A&D Company, Tokyo, Japan). Adding the liquid to the content of every amalgam cap-sule (glass powder and fiber) was done on a glass plate using a metal spatula within a 25-second mixing time (two drops of liquid for conventional resin modified glass ionomer and one drop for modified resin). Working time was considered 2 minutes for con-ventional and 3′45″ for resin modified glass ionomer. After placing the produced material in the mold, a glass slab was pressed against the mold to flatten the top surface. Setting time was considered 2′30″ for conventional glass ionomer. Samples were kept in an incubator for 24. The condition set by the incubator (Es 250, NUVE Company, Turkey) was the temperature of 37ºC and a relative humidity of 100%. Samples were thermo-cycled (Rika-kogyo, Hachoj, Japan) for 1000 cycles in water for 30 sec of dwell time at 5±2 and 55±2ºC. Thermo-cycling was done to stimulate clinical thermal stresses. Then samples were stored in deionized water at 37ºC in the incubator. The diametral tensile strength was measured after one week of storage in water. The diametral compression test was performed using Zwick/Roell universal testing machine (ZO20, Germany) with a crosshead speed of 2 mm/min fig. 2. The compressive load was placed by a flat plate against the side of the short cylindrical specimen. Diametral tensile strength was calculated from the following formula and expressed in MPa (11). Diametral tensile strength = 2P / πDt

Where *P* was the applied load (N), D as the diameter of the samples (mm) and t as the thickness of the samples (mm).

Statistical analysis was performed using SPSS software (version 11.5, Chicago, IL, USA). The Two-way ANOVA and Posthoc Tukey tests were used to compare the groups and a *p* value less than 0.05 was considered statistically significant.

## Results

**Figure 3:**
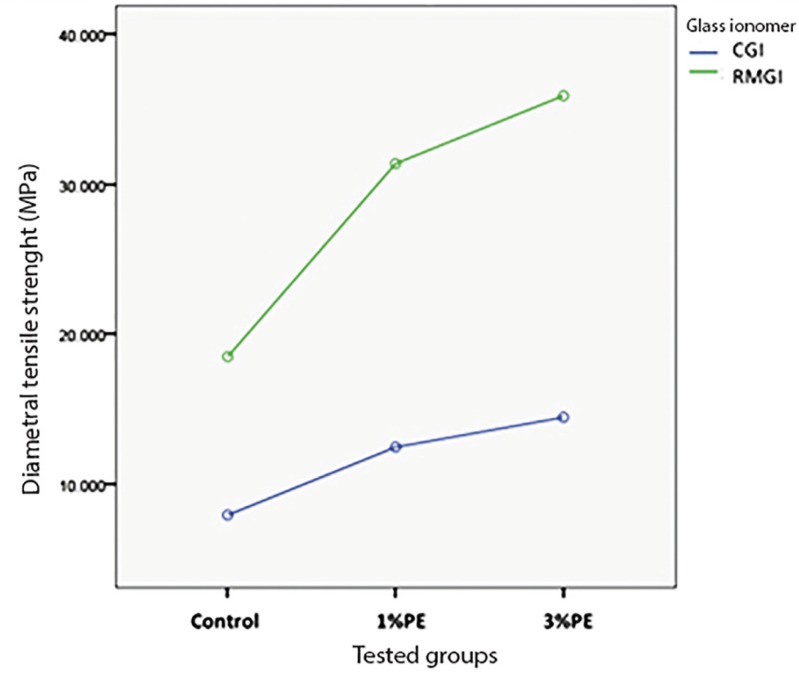
Diametral tensile strength of tested groups.

There was a significant difference between the two types of glass ionomers and polyethylene fiber (PE) containing groups (*p*<0.001) ( Table 1). The diametral tensile strength of each reinforced group was significantly higher than the associated control group ( Table 2). There was a significant difference between 1% and 3% wt reinforced conventional glass ionomer (CGI) groups (*p*<0.001). A significant difference was seen between 1% and 3% wt reinforced resin modified glass ionomer (RMGI) groups (*p*<0.001). The resin modified glass ionomer group was shown to be more affected by incorporation of the fiber than conventional glass ionomer group (Figs. 1-3).

**Table 1:**
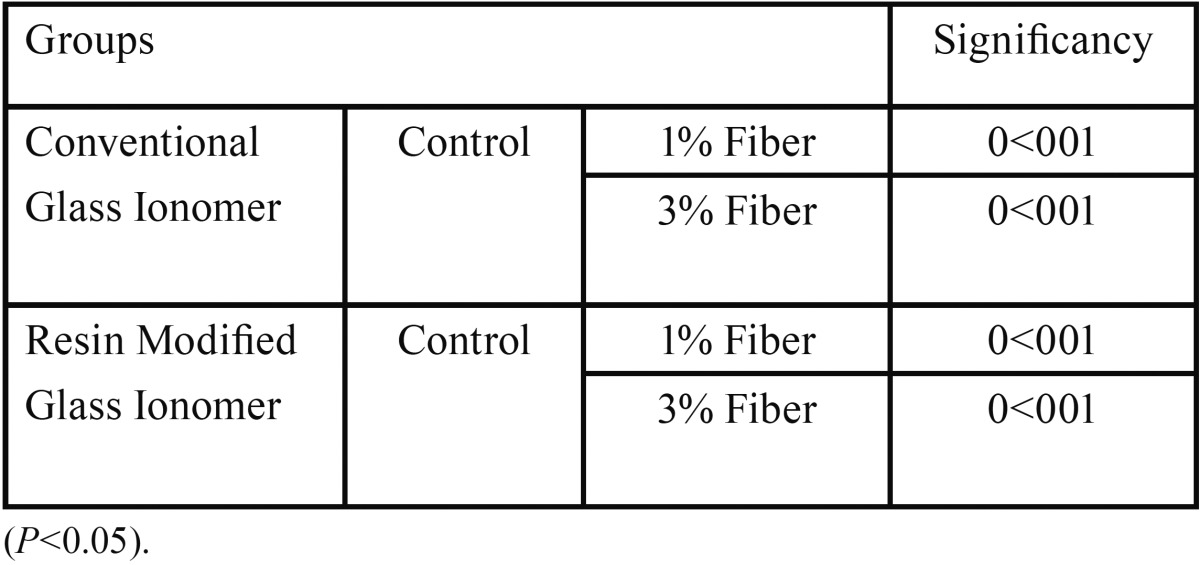
Multiple comparisons between resin modified and conventional glass ionomer groups.

**Table 2:**
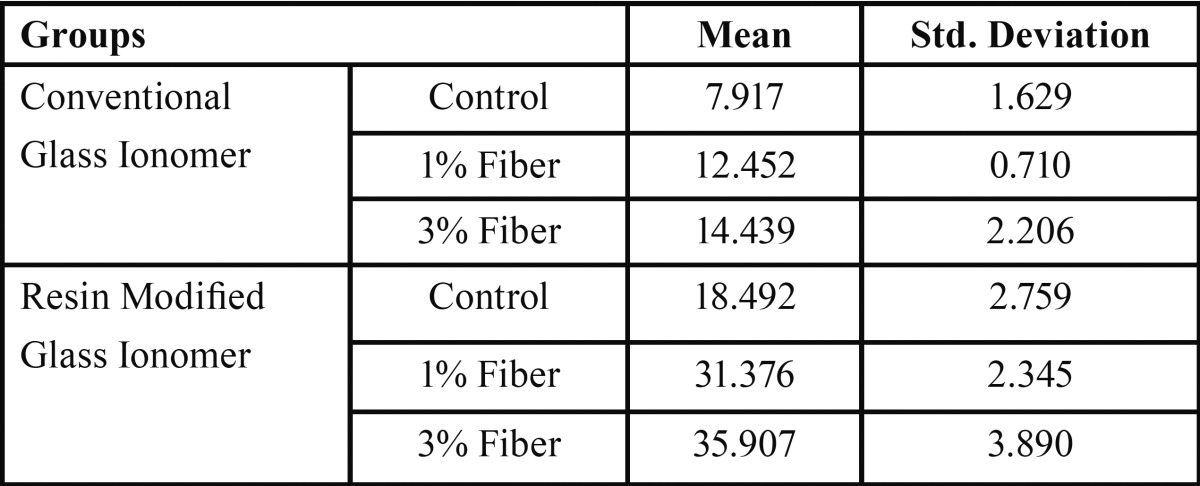
The mean value of diametral tensile strength (MPa) among different groups.

## Discussion

The use of glass ionomers as a restorative material is still questionable in many clinical areas. Reinforcements such as resin modification or metal-reinforcements have not still been satisfactory in clinical practice, especially in load-bearing areas (15). The effect of adding 10 and 30% wt of bioactive glasses (BAG) was evaluated on RMGI and CGI and it was reported that the compressive strength of the specimens decreased with an increase in the amount of BAG (7).

The 20% vol reactive glass and 60% mass glass fibers were also tested in other studies. Addition of 3 and 5% wt of short glass fibers to CGI was also evaluated in another study (11,12,16). The recent three reports denoted to desirable effects of the fibers on mechanical properties of CGI. In the present study, polyethylene fiber was used to reinforce CGI and RMGI and the used fibers were different from those used in previous studies regarding the material and loading. The fiber loading was 1 and 3% wt to avoid accidental changes in the surface smoothness of the restorative material and deterioration of mechanical properties.

Unlike glass fibers, polyethylene fibers have not yet been tested to be applied with glass ionomers, except in a study showing that glass and polyethylene fibers were more potent in reinforcing glass ionomers (14). It was demonstrated that the structural similarity of glass powder and glass fiber lead to a more desirable reinforcing effect, but Sharafeddin *et al.* found that polyethylene fibers had better effects (14).

In our study, polyethylene fiber was mixed with glass ionomers in two different concentrations in order to investigate their effect on diametral tensile strength. The present study revealed a significant increase in diametral tensile strength of both conventional and resin modified glass ionomers when mixed with 1 and 3% wt polyethylene fibers showing that fiber reinforcement may be beneficial to obtain stronger glass ionomers.

Flexural strength, flexural modulus and fracture toughness of the glass ionomer cement were previously determined by testing the material under both tensile strength and compressive loading (17). In 2004, the reports showed that the crosshead speed had a marked influence on the mechanical properties of the tested material (18). So we selected crosshead speed of 1 mm/min to perform the test.

We demonstrated that the tested materials presented an increase in diametral tensile strength as a result of fiber loading and storage time while storage time could affect the mechanical properties of the glassionomer. It was shown that the diametral tensile strength of resin modified glass ionomer increased in the period of 1 h to 1 week. This increase can be explained by the setting reaction of glass ionomer cements. Aluminum polycarboxylate which is more stable and improves the mechanical properties of the cement takes a mean of 24 h period to be formed (19). Glassionomers are susceptible to water dehydration and crazing during the initial setting reaction. The resultant micro cracks would act to initiate and facilitate crack propagation within the cement matrix during setting (20). Also, it was shown that glass ionomer cements tended to exhibit an increase in mechanical properties over the 24h period and to maintain a constant strength (21). Identical findings were reported by others (11). One week water storage is performed in our study to complete the strengthening of the setting. So we tested the specimens 24 h later.

There are also evidences of successful fiber incorporations to a composite resin (22). Short fibers (3 mm length) were added to experimental composite resin resulting into a significant increase in flexural strength and compressive loadbearing capacity (10,23-25). The effect of adding fibers to resin modified glass ionomer has rarely been investigated while in this study adding polyethylene fibers to RMGI lead to a prominent increase in diametral tensile strength. This increase was continued significantly as the fiber concentration increased from 1 to 3% wt. The effect of polyethylene fiber was more on RMGI than CGI. It was shown that polyethylene fiber could reinforce the RMGI more than CGI and cermet (9). The reinforcement of acrylic resin with polyethylene fiber was previously studied and was demonstrated that the fiber incorporation could effectively reduce the stress concentrations at stress-bearing areas (26). The main similarity of RMGI and composites and the main difference between RMGI and CGI were the presence of resin. Due to the good polyethylene/resin integration, adding polyethylene fiber to RMGI can be more effective than adding this type of fiber to CGI.

Polyethylene fibers are known to have a high tensile strength. The undesirable property of polyethylene in industry is its thermo-sensitivity (9). In oral cavity, polyethylene is not exposed to temperature in which thermal destruction occurs. In general, melting point of different types of polyethylene varies from 105 to 180°C.Thermo-cycling which simulates thermal changes and aging in oral cavity never reaches undesirable degrees in which polyethylene starts a degradation process. So the noticeable effect of the fiber in the present study might be referred to inherent high tensile strength of polyethylene.

Comparison between means of diametral tensile strength of tested specimens in this study and other authors showed that 1% polyethylene fiber could reinforce CGI more than 3% glass fiber and also 3% polyethylene fiber could be more potent than 5% glass fiber. Polyethylene fiber is known as a more flexible fiber than glass fiber (27).

Since fiber overload can be an obstacle in the way of reinforcement, it seems that lower percentages of polyethylene fiber combined with glass ionomers can reach same diametral tensile strength as higher percentages of glass fiber (13). So polyethylene might be a better choice than glass fiber. Further investigations are suggested with different kinds of polyethylene and different mixing percentages to evaluate them against each other and the glass fiber.

In conclusion, short polyethylene fibers especially with 3%wt could increase diametral tensile strength of conventional and resin modified glass ionomer which is clinically important for clinical use of glass ionomers especially in load bearing areas.
